# A New Pleistocene Hominin Tracksite from the Cape South Coast, South Africa

**DOI:** 10.1038/s41598-018-22059-5

**Published:** 2018-02-28

**Authors:** Charles W. Helm, Richard T. McCrea, Hayley C. Cawthra, Martin G. Lockley, Richard M. Cowling, Curtis W. Marean, Guy H. H. Thesen, Tammy S. Pigeon, Sinèad Hattingh

**Affiliations:** 1 Peace Region Palaeontology Research Centre, Box 1540, Tumbler Ridge, British Columbia V0C 2W0 Canada; 2 African Centre for Coastal Palaeoscience, Nelson Mandela University, PO Box 77000, Port Elizabeth, 6031 South Africa; 3 Marine Geoscience Unit, Council for Geoscience, PO Box 572, Bellville, 7535 South Africa; 4 Dinosaur Trackers Research Group, Campus Box 172, University of Colorado Denver, PO Box 173364, Denver, 80217-3364 USA; 5 Institute of Human Origins, School of Human Evolution and Social Change, Arizona State University, PO Box 874101, Tempe, AZ 85287-4101 USA; 6 South African Spelaeological Association, PO Box 30233, Tokai, 7966 South Africa

## Abstract

A Late Pleistocene hominin tracksite has been identified in coastal aeolianite rocks on the Cape south coast of South Africa, an area of great significance for the emergence of modern humans. The tracks are in the form of natural casts and occur on the ceiling and side walls of a ten-metre long cave. Preservation of tracks is of variable quality. Up to forty hominin tracks are evident. Up to thirty-five hominin tracks occur on a single bedding plane, with potential for the exposure of further tracks. Five tracks are apparent on a second hominin track-bearing bedding plane. A number of individuals made the tracks while moving down a dune surface. A geological investigation at the site and stratigraphic comparison to published geochronological studies from this area suggest that the tracks are ~90 ka in age. If this is the case, the shoreline at the time would have been approximately 2 km distant. This is the first reported hominin tracksite from this time period. It adds to the relatively sparse global record of early hominin tracks, and represents the largest and best preserved archive of Late Pleistocene hominin tracks found to date. The tracks were probably made by *Homo sapiens*.

## Introduction

The Cape south coast of South Africa has one of the richest Middle Stone Age archaeological records in the world, and is of high importance to modern human origins. Sea cliff caves, rock shelters, and open-air sites on the exposed landscape hold archives of early humans^1,2^. The emergence of modern humans in southern Africa is a subject of considerable interest, and the Cape south coast figures prominently in this research^2–6^. These sites provide early evidence for art and jewelry^3,4^, stone tool heat treatment^7^, microlithic technology^8^, and the first systematic use of seafood in the human diet^1^. In addition, inferences on palaeoclimate^9,10^ and palaeolandscape^11,12^ suggest that factors such as the confluence of ocean currents, the broad continental shelf and sweeping plain, and vegetation supported by the geological substrate, may have been conducive to the survival of coastal Pleistocene hominin communities.

The potential for ichnology to contribute to such research has been limited by the sparse record of early hominin tracksites. The most comprehensive summaries of such sites include a table of 63 previously known hominin tracksites^13^, a table of 44 sites^14^, and an ichnological summary of major events in hominin evolution^15^. Based on these tables, known early hominin tracksites prior to the discovery of the site we describe, in order of decreasing age, and with purported trackmaker, included:

- Laetoli, Tanzania (~3,660 ka): *Australopithecus afarensis*
- Ileret, Kenya (~1,500 ka): *Homo erectus*
- Koobi Fora, Kenya (~1,400 ka): *Homo erectus*
- Happisburgh, United Kingdom (~1,000–780 ka): *Homo antecessor*
- Roccamonfina Volcano, Italy (~385–325 ka): *Homo heidelbergensis*
- Terra Amata, France (~300 ka): *Homo erectus*
- Nahoon, South Africa (~126 ka): *Homo sapiens sapiens*
- Langebaan, South Africa (~117 ka): *Homo sapiens sapiens*
- Vârtop Cave, Romania (constrained to 62–97 ka): *Homo neanderthalensis*
- Theopetra Cave, Greece (~46 ka): *Homo sapiens sapiens*.
  Thereafter many more recent sites follow, from all continents except Antarctica. We note that the tables of Lockley *et al*.^13^ and Bennett and Morse^14^ do not include the enigmatic, undated “Queen Nzinga’s footprints” at Pungo Andongo in Angola^16^.
  Rigour is required in the attribution of tracks to hominins. Tuttle^17^ developed criteria for the identification of hominin tracks:
- the hallux (big toe) is aligned with the four lateral toes, which are short and straight
- the tip of the hallux is bulbous, not tapered
- the tips of the hallux and adjacent second and third toes do not project markedly beyond one another
- a prominent medial longitudinal arch is evident.

Published research on South African Pleistocene trackways has largely centred on the two above-mentioned hominin tracksites (Nahoon and Langebaan) and nearby associated vertebrate tracks^18,19^, plus on elephant trackways^20,21^. Both South African hominin tracksites contained three human tracks. The first site was discovered in 1964 at Nahoon, 600 km to the east of the site we describe^22^. It collapsed soon after its discovery. The tracks were recovered, and are housed in the East London Museum^19,23^. The second site, near Langebaan, 400 km to the west of the site we describe, was discovered in 1997^19,24^. The tracks are housed in the Iziko South Africa Museum, Cape Town. Both these sets of tracks were found in aeolianite deposits which date to the last interglacial period, Marine Isotope Stage (MIS) 5e. The Nahoon site has been dated to ~126 ka, and the Langebaan site to ~117 ka^19^. They have been thought to represent the first known tracks made by our own subspecies, *Homo sapiens sapiens*. However, this assumption was made prior to the description of a second southern African hominin species (*Homo naledi*) from skeletal material from the Rising Star Cave^25^ (more than 750 km north of these sites, and a linear distance of more than 1000 km from the site we describe) and its subsequent dating, which provides an estimated minimum age of 236 ka^26^.

The general prevalence and significance of MIS 5 aeolianites along the Cape south coast has been discussed extensively in the literature^19,27,28^. The hominin tracksite we describe is the first on the Southern Coastal Plain, and is situated between the Nahoon and Langebaan sites.

**Figure 1:**
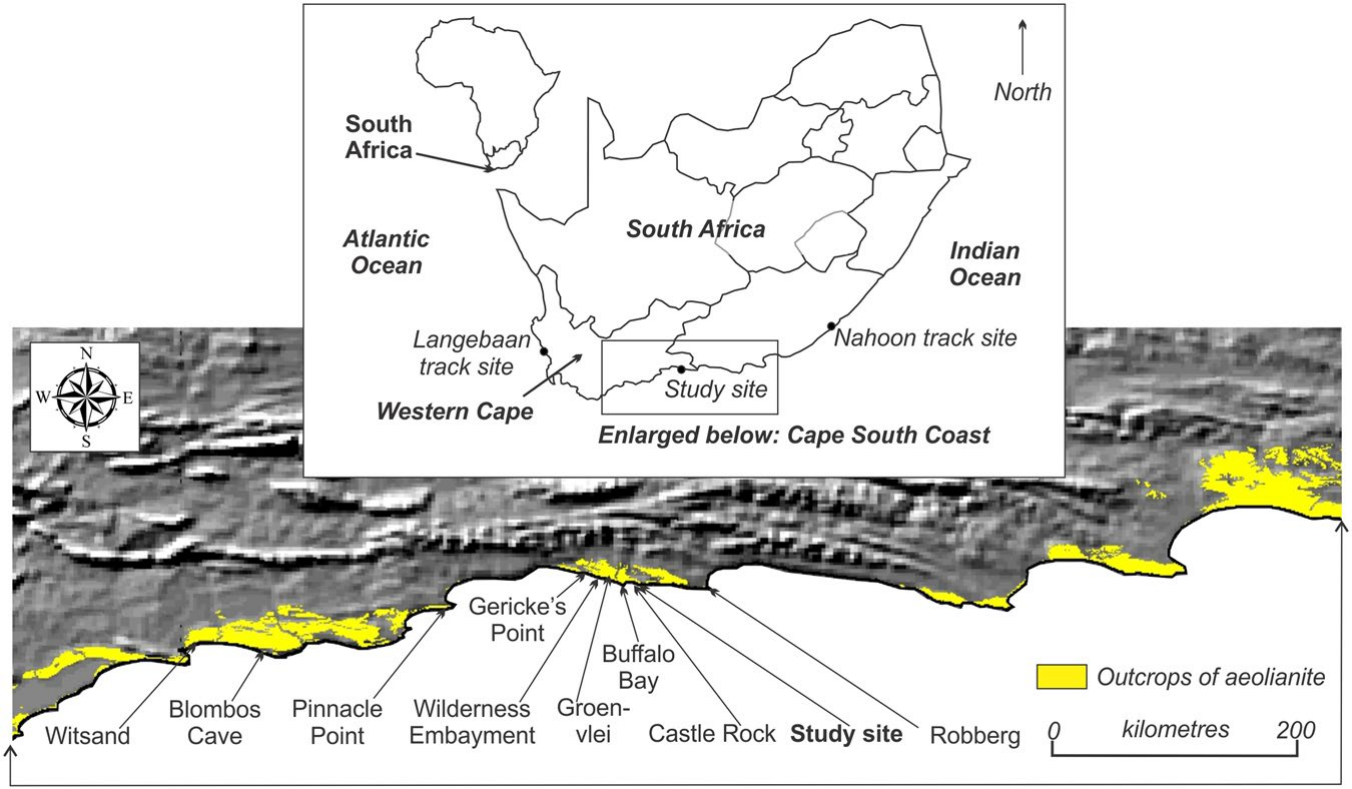
Map of South Africa and the Cape south coast, showing sites discussed, created by co-author HC. SRTM (USGS, 2004, Shuttle Radar Topography Mission, 1 Arc Second scene SRTM_u03_n008e004, Unfilled Unfinished 2.0, Global Land Cover Facility, University of Maryland, College Park, Maryland, February 2000) 90 m resolution elevation data (http://www.cgiar-csi.org/data/srtm-90m-digital-elevation-database-v4-1) was overlain by Council for Geoscience 1:250,000 scale geological units of the Bredasdorp Group using ArcGIS software. The final figure was produced in CorelDrawX7 version 17.6.0.1021 (https://www.coreldraw.com/en/pages/free-download/).

**Figure 2:**
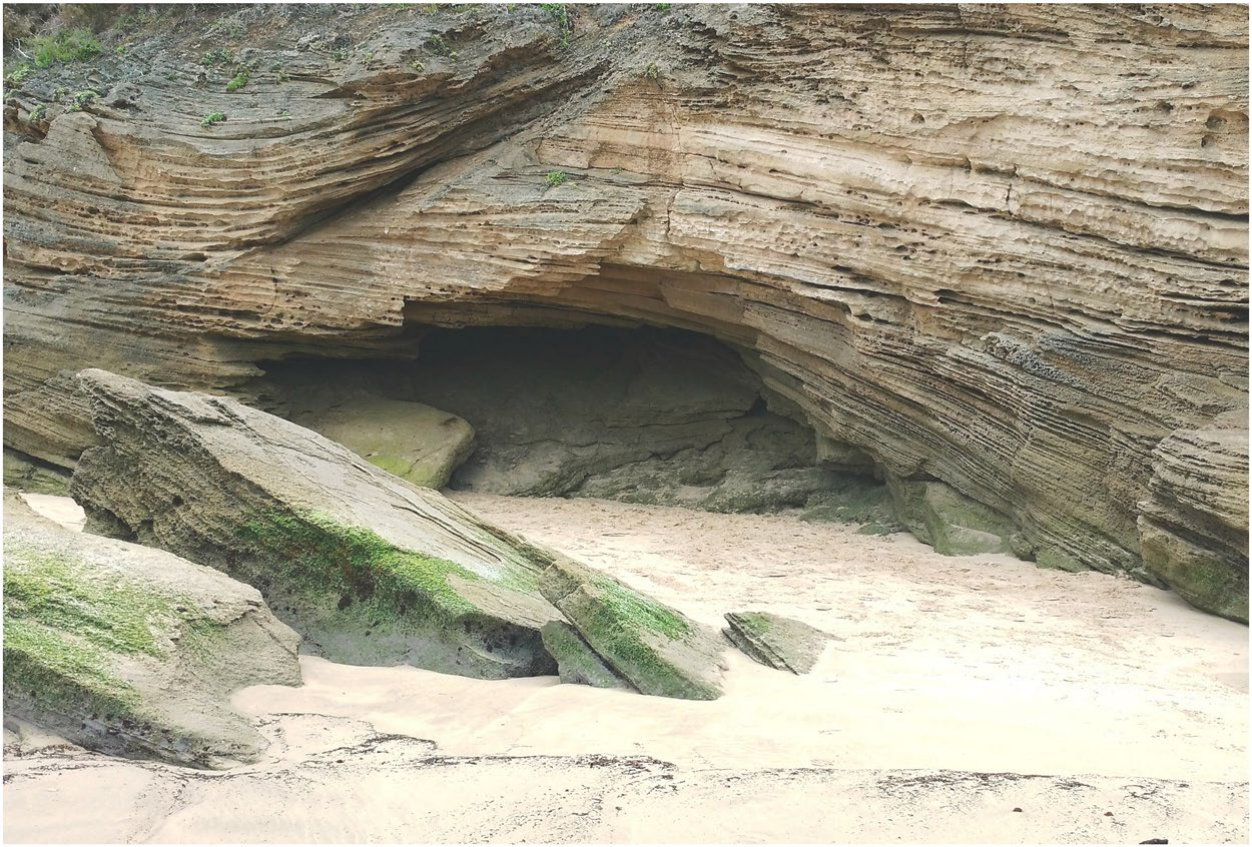
Entrance of cave containing hominin tracks, viewed from the intertidal zone (photograph by co-author ML).

The hominin tracks reported here were discovered as part of a ground survey by the senior author along a 275 km stretch of coastline from Witsand in the west to Robberg in the east, undertaken between 2007 and 2016 (Fig. 1). Over 100 Late Pleistocene vertebrate tracksites were identified in coastal aeolianites, and in 2016 natural cast tracks on the ceiling of a ten-metre long cave (Fig. 2) (see Supplementary Figs S2–S5) were identified as human in origin. In 2017 further hominin tracks were identified in this cave on a lower layer. The focus of this paper is to describe these tracks and to briefly place them in their sedimentary and palaeoecological context.

## Geological setting

The Late Pleistocene tracks reported here occur in coastal aeolianites, or fossil dune systems, which extend intermittently along much of the South African coastline and are best exposed in embayments on or near the shoreline^21^. Notable phases of dune construction in the current above-water record appear to be associated with regressive phases that followed sea-level high stands^27,28^, although deposits preserved on the continental shelf were associated with both transgressive and regressive sea-levelevents^29^.

Less than a quarter of the Cape south coast comprises aeolianite exposures^30^; the remainder of the geological substrate of the Southern Coastal Plain is dominated by Palaeozoic quartzite, sandstone and shale exposures of the Cape Supergroup, granite exposures of the Cape Granite Suite, and expanses of beach and unconsolidated Holocene beach and dune sediments. Aeolianites along the South African coast are sensitive barometers of fluctuations of palaeo-environmental dynamics, providing records of their orientation, geometry, palaeontology and archaeological content^21^. This record is enhanced by the identification of the tracks reported here.

The Late Pleistocene coastal trackways, including the hominin tracksite, occur in the Waenhuiskrans Formation, which belongs to the Bredasdorp Group^31^. Numerous dating studies have been performed on rocks of the Waenhuiskrans Formation through optically stimulated luminescence (OSL), Thermally Transferred OSL (TT-OSL) and amino acid racemisation (AAR) dating^20,28,29,32,33^. The majority of these dated aeolianites are from MIS 6 – MIS 5b, with two occurrences of MIS 11. The preservation bias to interglacials along the modern Southern Coastal Plain is, however, a function of the present high sea level regime^34^.

Quaternary tectonic activity has been considered minimal on the Cape south coast^35,36^, and the *in situ* bedding planes of the aeolianite hosting the hominin tracks lie close to their original angle of deposition, which is consistent with the angle of repose of wind-blown sands (~10°–30°)^21^.

Globally, the significance of coastal barrier systems and associated aeolianites in palaeo-environmental studies is well established^37^. They record the geomorphic evolution of coastal margins^38–40^ and coastal geomorphological responses to Pleistocene sea-level change^41^. In the South African context they are key to understanding Middle and Later Stone Age archaeological sites and the associated palaeo-environments that our ancestors occupied^1,20,42^.

## Methods

Geological context was determined by standard field mapping of the exposure and outcrop. Primary sedimentary structures, including dip direction, were recorded for the construction of wind roses, samples were obtained for transmitted light microscopy, and the exposures were compared to adjacent deposits reported in previous investigations. Facies were described through analysis of the sediment grain characteristics and carbonate diagenetic signature. Stratigraphic comparison was performed to a dated site ~1 km distant (Castle Rock), and to the seaward cordon of the Wilderness Embayment (Fig. 1).

During the course of five site visits in 2016, a grid system was employed to create a sketch map of the track-bearing surfaces, along with a numbering system for the tracks. Photographs, Global Positioning System readings and compass bearings were obtained. Track and trackway measurements were recorded, and a cave survey was performed (see Supplementary Fig. S1). The tracks on the lower layer were measured in 2017.

**Figure 3:**
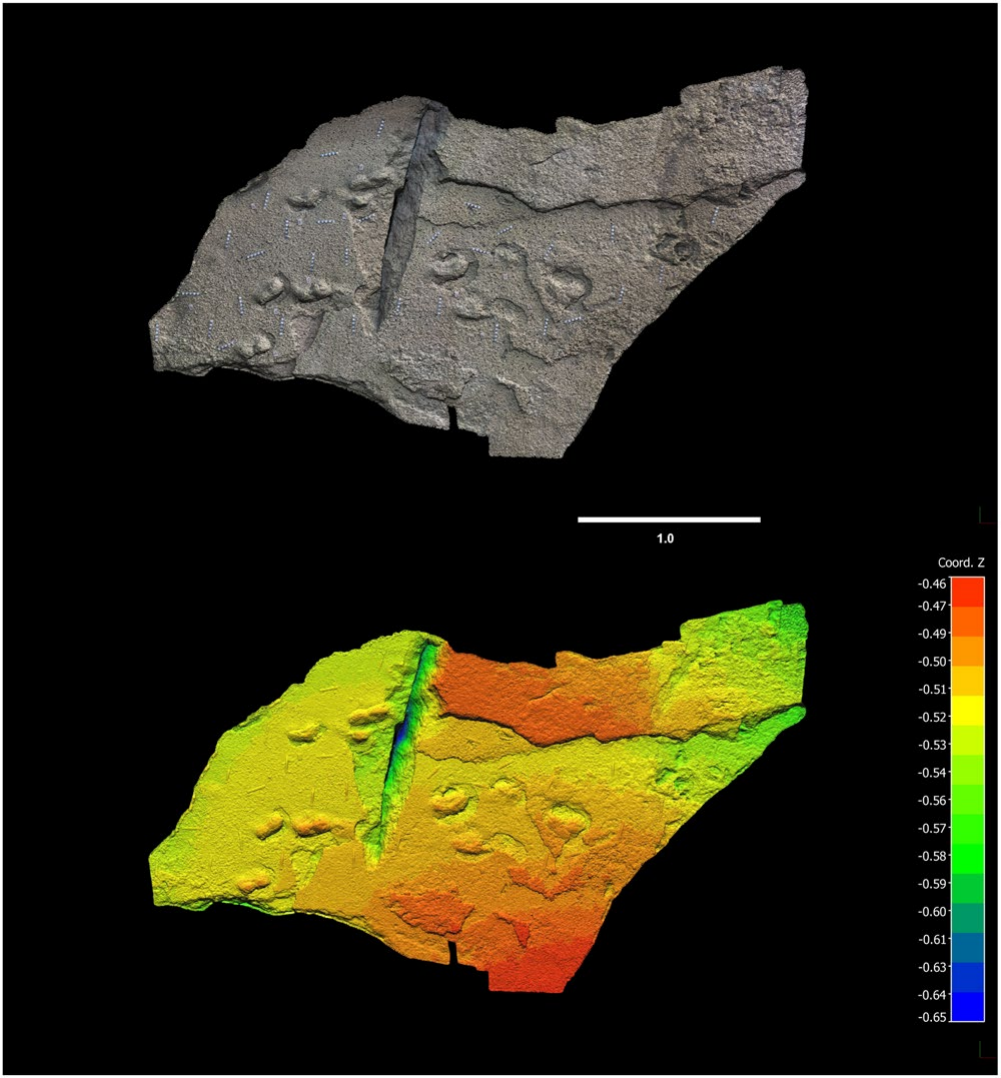
Photogrammetry of hominin tracks, southern surface. Top: photogrammetry mesh. Bottom: photogrammetry colour mesh. 3D model was generated with Agisoft Photoscan Professional (v. 1.0.4) using 200 images from a Canon PowerShot ELFPH 340 HS (Focal length 4.5 mm; resolution 4608 × 3456; pixel size of 1.33853 × 1.33853 um). Photos were taken average 0.54 metres from the surface. The surface model error is 0.131213pix. The final images presented here were rendered using CloudCompare (v.2.6.3.beta). Horizontal scale bar, representing *x* and *y* axes, is in metres. Vertical scale, representing *z* axis, is in metres.

**Figure 4:**
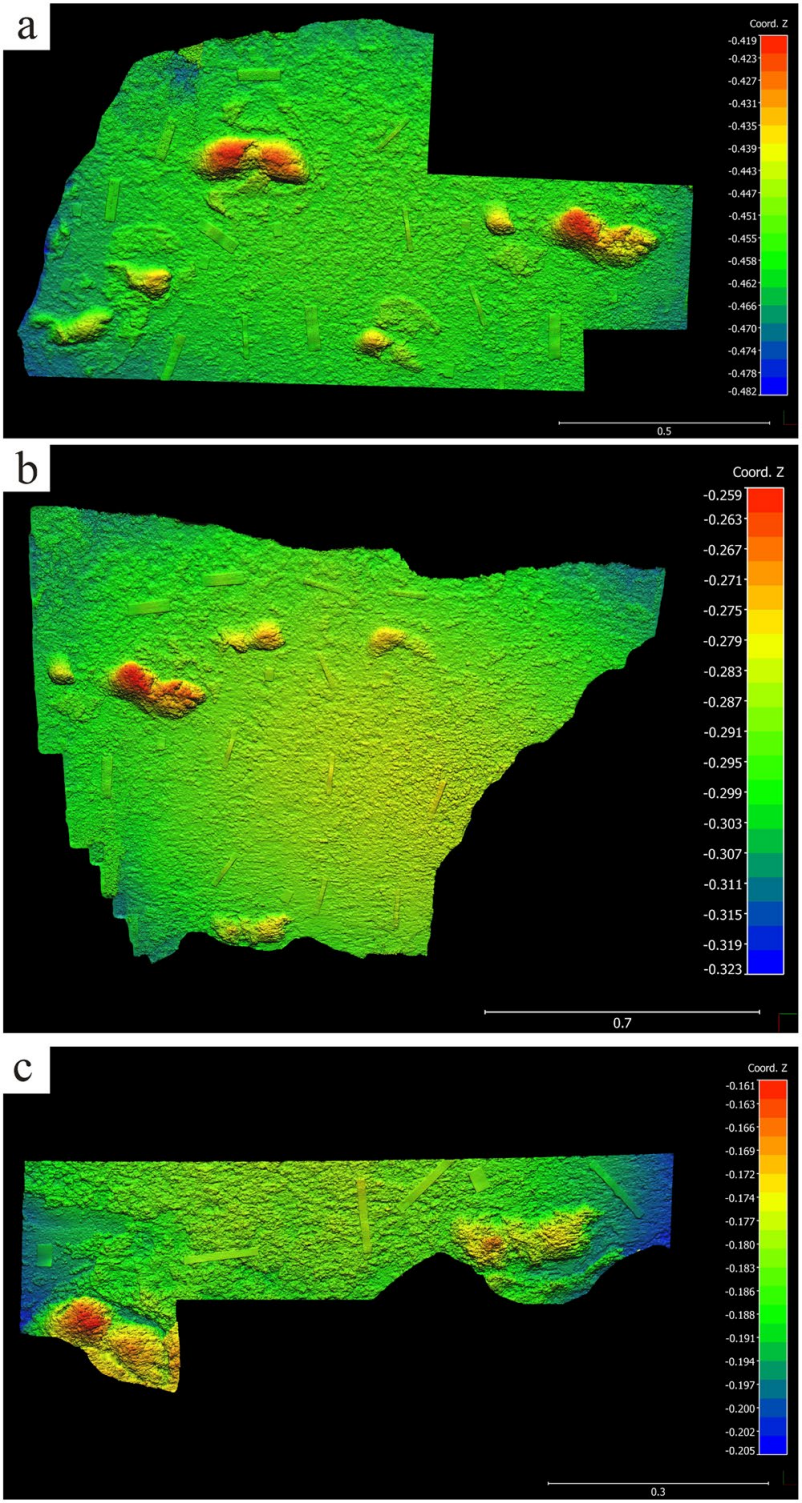
Photogrammetry of hominin tracks, northern surface. (**a**) Photogrammetry colour mesh of northern surface, proximal. 3D model was generated with Agisoft Photoscan Professional (v. 1.0.4) using 263 images from a Canon PowerShot ELFPH 340 HS (Focal lengths of 4.5 mm and 4.881 mm; resolution 4608 × 3456; pixel size of 1.33853 × 1.33853 um). Photos were taken average 0.41 metres from the surface. The surface model error is 0.150518 pix. The final images presented here were rendered using CloudCompare (v.2.6.3.beta). (**b**) Photogrammetry colour mesh of northern surface, distal. 3D model was generated with Agisoft Photoscan Professional (v. 1.0.4) using 198 images from a Canon PowerShot ELFPH 340 HS (Focal length 4.5 mm; resolution 4608 × 3456; pixel size of 1.33853 × 1.33853 um). Photos were taken average 0.45 metres from the surface. The surface model error is 0.132266pix. The final images presented here were rendered using CloudCompare (v.2.6.3.beta). The large track in figure is the same as the track on the far right of (**a**). (**c**) Photogrammetry colour mesh, detail of tracks in northern surface beside east wall. 3D model was generated with Agisoft Photoscan Professional (v. 1.0.4) using 155 images from a Canon PowerShot ELFPH 340 HS (Focal lengths of 4.5 mm – 5.082; resolution 4608 × 3456; pixel size of 1.33853 × 1.33853 um). Photos were taken average 0.17 metres from the surface. The surface model error is 0.153466pix. The final image presented here was rendered using CloudCompare (v.2.6.3.beta). The footprint at the far right of this figure is the same as the bottom-most print in (**b**). For (**a**,**b**) and (**c**), horizontal scale bar, representing *x* and *y* axes, is in metres. Vertical scale, representing *z* axis, is in metres.

**Figure 5:**
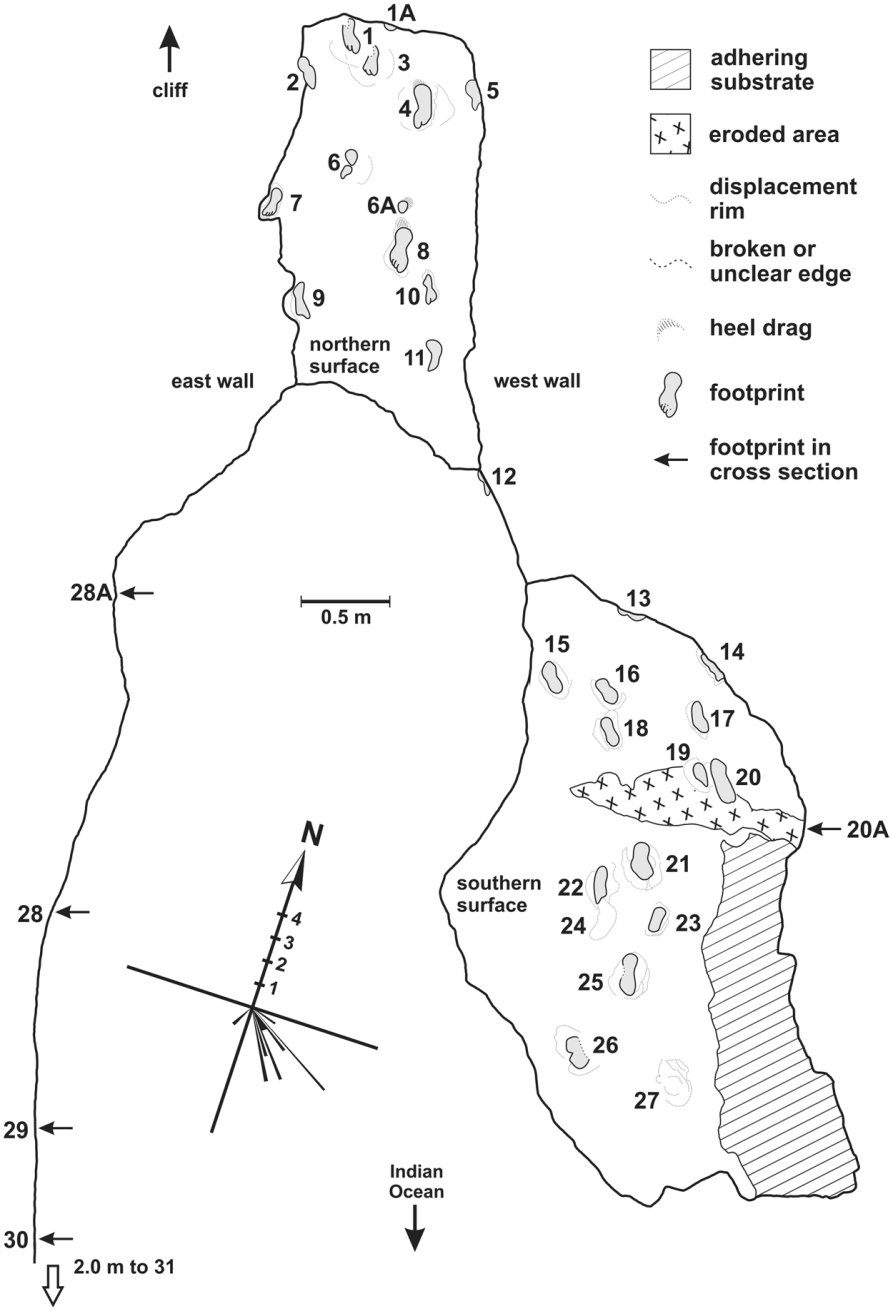
Map of track-bearing surface with rose diagram of track orientation, created by co-authors TP and RM, using Corel Draw X7, version 17.6.0.1021 (https://www.coreldraw.com/en/pages/free-download/).

Photogrammetry was performed using a Canon PowerShot ELFPH 340 HS camera. Point clouds and digital terrain models were compiled using Agisoft Photoscan Professional (v.1.0.4) and colour topographic profiles were created with CloudCompare (v.2.6.3.beta) (Figs 3 and 4). A track surface map was produced (Fig. 5).

The site was reported to Heritage Western Cape. Precise locality information for this site is reposited at the African Centre for Coastal Palaeoscience.

### Data and materials availability

As mentioned in the manuscript, locality information for this site is reposited at the African Centre for Coastal Palaeoscience. Site co-ordinates will not be publicly released until appropriate site protection measures have been undertaken, with the involvement of Heritage Western Cape. Data and materials will be released to *bona fide* researchers through the corresponding author.

## Results

**Figure 6:**
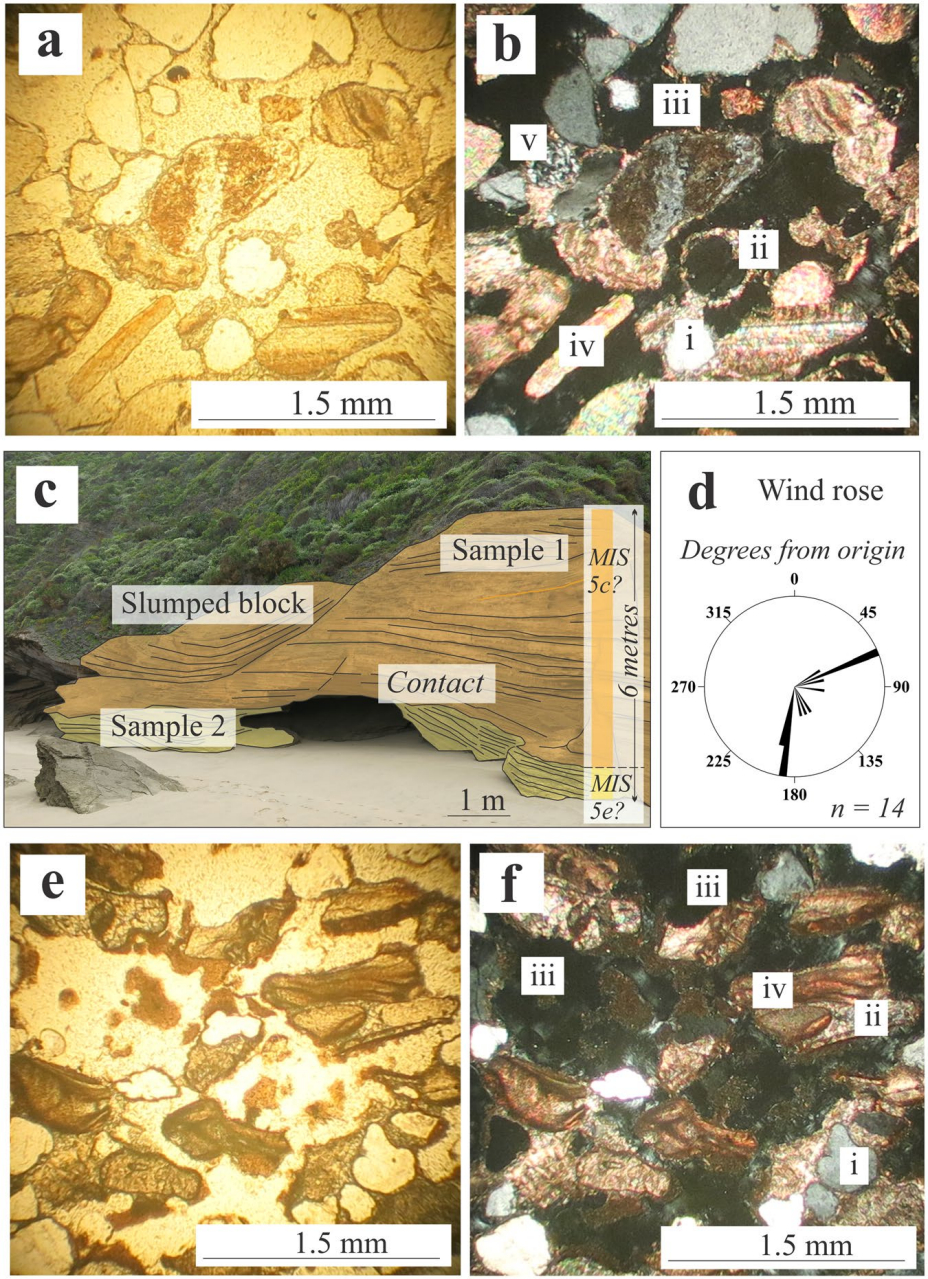
Sediment analysis (images by co-author HC). (**a**) Plane-polarised light petrographic microscope image of Sample 1. (**b**) Cross-polarised light petrographic microscope image of Sample 1. **(c)** Sample context at the site under investigation. The area of Sample 1 is characterised by thicker planar beds and the basal part of the outcrop from where Sample 2 was obtained consists of more finely laminated foresets. Cave incision has taken place at the contact between these two aeolian facies, but the age is likely consistent through the sequence. (**d**) Wind rose derived from dip and strike measurements on bedding planes. (**e**) Plane-polarised light petrographic microscope image of Sample 2. (**f**) Cross- polarised light petrographic microscope image of Sample 2. In (**b**) and (**f**), i is a rounded quartz clast, ii is the blocky calcite spar cement which fringes grain boundaries. iii is a void or unfilled pore space, iv is a shell fragment and v is a lithic fragment which constitutes the matrix.

The local geology is characterised by a steep coastal cliff, made up of moderately-bedded, consolidated aeolianite. These aeolianite deposits are nested against an embayment carved into Palaeozoic quartzite. Aeolian foreset beds at the site vary in thickness, from 20 cm in the upper section of the cliff, to finely laminated layers near the cave entrance (2.5–5 cm) and up to 10 cm near the base of the succession. Incision of the overhang has taken place where the finely laminated beds occur and along the contact which separates foresets of different thickness, compaction and orientation. ‘Negative’/eroded laminae alternate between ‘positive’/protruding morphologic foreset beds on the cliff face where preferential weathering of less well-cemented forests has taken place (Fig. 6).

Dip and strike measurements on foresets (n = 14) indicate that these deposits were laid down by prevailing south-southwesterly and northeasterly winds. In thin section and using transmitted light microscopy, the composition of the clasts was revealed to consist of quartz, feldspar, lithic fragments, bivalve and gastropod fragments, foraminiferal tests and heavy minerals (Fig. 6). The dominant grain size is medium sand and the clasts are moderately- to well sorted. The sedimentary clasts are bound by calcium carbonate cement, which dominates the grain boundaries. This cement is made up of blocky- to drusy calcite spar. Approximately 30% of these deposits are made up of unfilled pores.

The tracksite reported here is located ~1 km from the outcrop at Castle Rock, and at the same elevation. The two sites exhibit comparable stratigraphy. The Bateman *et al*.^28^ chronology was determined by OSL, in which the seaward cordon was sampled and dated at Castle Rock (89 ± 6 ka), Groenvlei (91 ± 5 ka), Gericke’s Point (92 ± 5 ka) and Buffalo Bay (86 ± 5 ka, 91 ± 5 ka) (Fig. 1). Based on stratigraphic comparison to these dated sites, where 90 ka deposits overlie a 125–130 ka unit^28^, we consider the hominin track-bearing layers to be part of the younger (MIS 5c) outcrops, which are well-documented and described in Wilderness Embayment stratigraphy^28^. The suggested case for the tracksite we describe is that a ~90 ka sequence overlies a basal MIS 5e succession, and we propose that the aeolianite outcrops along the intertidal zone below the coastal cliffs at the tracksite can be correlated to this MIS 5e age. The incision of the coastal cave where the footprints are revealed on its ceiling took place where the aeolian foresets change in character, but this is close to the geological contact between these proposed MIS 5e units (which are dominant in the present intertidal zone) and stratigraphically higher MIS 5c units (which make up the coastal cliffs). We therefore propose a likely age of ~90 ka (MIS 5c) for the hominin tracks, within a sequence made up of two depositional events (basal MIS 5e overlain by MIS 5c) (Fig. 6).

Two main track-bearing surfaces are apparent on a single bedding plane, which forms much of the cave ceiling. The southern surface occurs towards the mouth of the cave. The northern surface occurs within the inner portion of the cave. Both surfaces show the tracks as natural casts (convex hyporelief). The two surfaces present differences in footprint preservation and morphology. Further tracks appear in sagittal and cross section in the lateral walls. Up to thirty-five complete or partial tracks were recorded. A further hominin track-bearing layer is evident in the south-eastern end of the cave, 31 cm below the main layer. Five tracks are evident on this lower layer as natural casts. In addition to these two track-bearing levels, which are the focus of the present study, at least three additional tetrapod track-bearing levels occur above and below the levels containing the hominin tracks.

**Figure 7:**
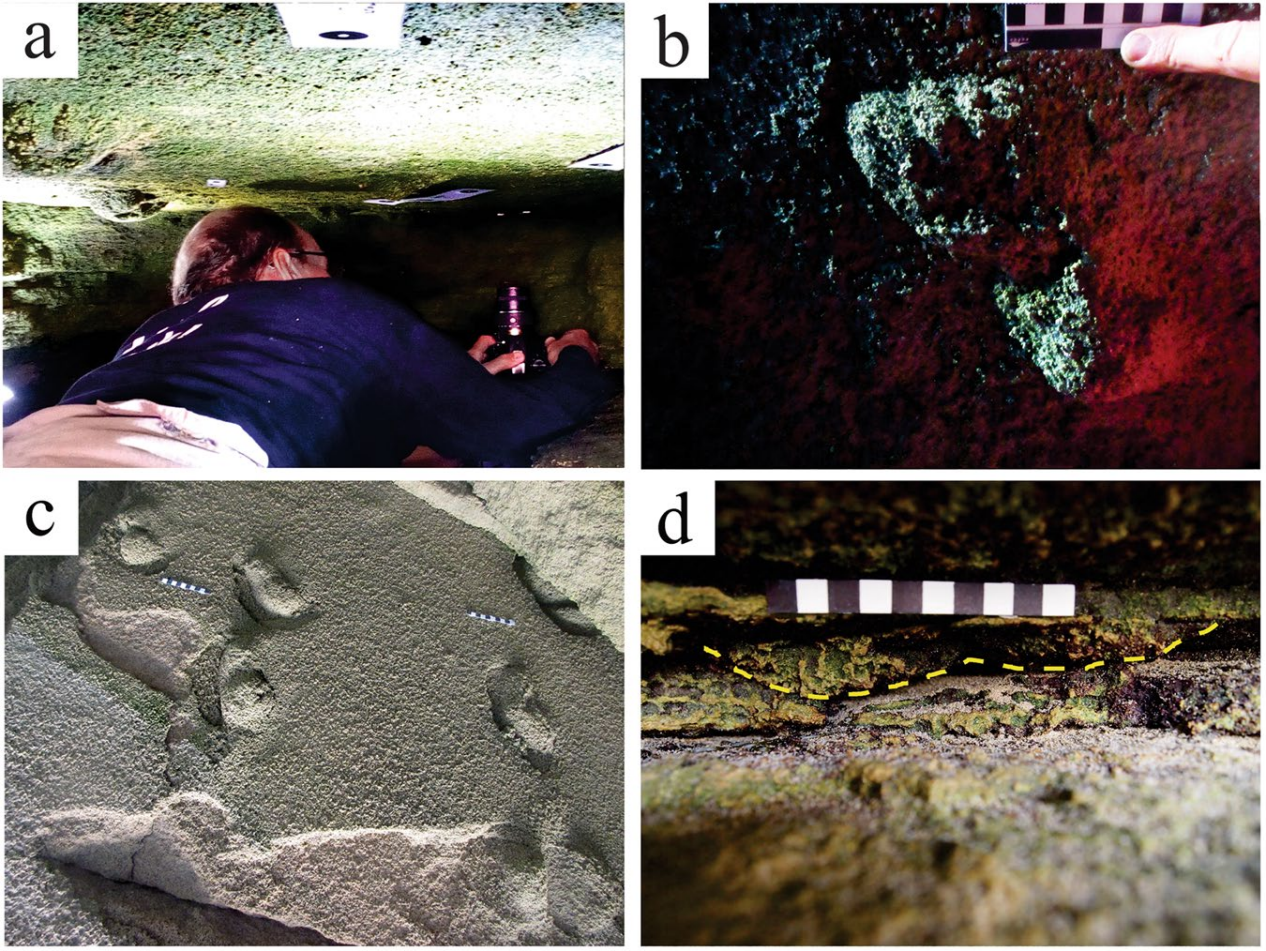
Photographs of hominin tracks. (**a**) Documenting natural cast tracks on the northern track-bearing surface (photograph by co-author SH). (**b**) Left natural cast track (track 8) on ceiling of northern surface showing hallux and lateral digit impressions and medial longitudinal arch, using natural light; scale bar = 10 cm (photograph by senior author CH). (**c**) Hominin natural cast tracks on ceiling of southern surface, surrounded by sediment displacement rims. Scale bars = 10 cm (photograph by senior author CH). (**d**) Natural cast track in sagittal section (outlined) in east wall; scale bar = 10 cm (photograph by co-author GT).

The cave floor lies near the spring high tide level, and its deeper (northern) portion is relatively confined, with a maximum distance between floor and ceiling of 50 cm, and distances of as little as 23 cm between floor and ceiling beside the lateral walls (Fig. 7a) (see Supplementary Figs S4 and S5). The natural cast tracks vary in their degree of preservation. Many are partially eroded. They extend to all edges of the track-bearing surfaces, and evidently beyond into the surrounding outcrop.

The thickness of the main track-bearing layer measures a minimum of 2.5 cm in the east wall, and a maximum of 5.8 cm in the west wall, west of which its upper surface becomes fused with the layer above it, with a thickness of up to 18 cm. The two main track-bearing surfaces are separated by an interval of 60 cm of horizontal surface at their closest approximation. Nine of the tracks on the northern surface exhibit digit casts (e.g. Figure 7b).

The tracks on the southern surface appear to have been made in a softer substrate, which rarely preserves digit casts. Sediment deformation and downslope displacement rims characterize many of the tracks on this surface (Fig. 7c). The tracks that are closest to the mouth of the cave are the most eroded and incomplete.

A third set of track casts is seen in sagittal section in the east wall of the cave on the same bedding plane – measurements indicate a pace length, orientation, and outline consistent with the other tracks (Fig. 7d). A small area of the main track-bearing surface is exposed at the far south-eastern end of the cave, and is only visible when this area has been scoured free of sand. It contains one natural cast in the shape of a hominin track.

The tracks are oriented in a downslope direction in a strongly unimodal orientation, as evidenced in a rose diagram (Fig. 5). The dip angle of the track-bearing bedding plane is 20°, consistent with a typical angle of repose of a dune slope^21^. Downslope sediment displacement rims are consistent with this geometry, and the thickness of foresets removed by erosion increases from 50 cm in the area below the northern surface to 90 cm in the area below the southern surface, over a downslope distance of 4 m, thus indicating a downslope thickening of the removed beds. The distance between the most proximal and most distal tracks is almost 10 metres.

Many tracks exhibit deep heel impressions. One trackway contains footprints that are clearly larger than the others (maximum length 27 cm with heel drag or 23 cm without heel drag; maximum width 10.5 cm; maximum depth 5.5 cm), while the rest are smaller (often ~17 cm long) (Table 1).

**Table 1:** details of tracks on main track-bearing layer (01–31) and lower layer (L01–L05).

Track	Length (cm)	Width (cm)	Depth (cm)	Toe traces	Left/right	Displace-ment rim	Comments
01	17	7	3	I, II	L	Y	Part of heel is absent
01A						Y	Anterior portion of displacement rim
02	18		3.5				At junction with east wall, deep heel
03	16	7.5		I, II	L	Y	Part of heel is absent
04	27 with heel drag, 23 without	10.5	5.5 (heel); 4 (ball)	I	R	Y (large)	
05	19			I?	R?		At junction with west wall
06	16.5	6.5		I	R	Y	
06A	6.5	6.5					Part of composite track?
07	19	7.5		I, II, III, IV	R		Deep heel, toes are surrounded by rock
08	27 with heel drag, 23 without	10	5 (heel); 4 (ball)	I, II, III, IV	L		
09	20	7		I	L		Near junction with east wall
10	15?	7?		I?	R?		Poor preservation, truncated?
11	16?	6?					Poor preservation
12	18			I?	R?		In cross section in west wall
13	17 or greater				R?		In cross section in west wall
14	20						In west wall
15	17.5	8.5			L?	Y	
16	17	8				Y	
17	18	9			L?	Y	
18	18.5	9				Y	
19	12	7				Y	Partial, possibly composite
20	22	10			L?	Y	
20A			2.5				At junction with west wall
21	20	10			L?	Y	
22	17	7				Y	Poor preservation
23	15	6				Y	Poor preservation
24	20	10					No natural cast, only footprint shape
25	21.5	9.5			L?	Y	
26						Y	Eroded remnant
27						Y	Eroded remnant
28A	18		4				In sagittal section in east wall
28	18		2.5				In sagittal section in east wall
29	21		3				In sagittal section in east wall
30	16		1.5				In sagittal section in east wall
31	19	7	1.5		L		Eroded remnant
L01	19		6				In cross section, east wall, lower layer
L02							Eroded remnant on lower layer
L03	12	5	2.5				Smaller track on lower layer
L04							Small, inaccessible unmeasurable
L05	16	7	1.5	I	L	Y (large)	On lower layer

The lower track-bearing layer is exposed at the southeastern end of the cave, ~31 cm below the main track-bearing layer. These tracks are only visible when the cave mouth area has been scoured free of sand. The maximum length of this surface is ~220 cm, with a maximum width of ~60 cm. Up to five tracks are evident, with an apparent unimodal orientation that is directed more southwest than the tracks on the main track surfaces. In this area there is a maximum space of 27 cm between the layer containing the track casts and the surface below, and a minimum space of 3 cm. Hominin track morphology could therefore only be reliably confirmed for the three most accessible tracks on this layer, and only basic track measurements could be obtained (Table 1). A large sediment displacement rim was noted around one of these tracks along with a faint toe trace.

This lower track-bearing layer can be followed along the east wall, north wall and west wall of the cave. The distance between the main track-bearing layer and the lower track-bearing layer varies from 25–35 cm. At least four moderately large tracks are evident in the west wall in this layer (diameter 13–17 cm, depth 4–5 cm) but cannot be identified further at present.

Two small even-toed ungulate tracks are visible on the southern surface of the main hominin track-bearing layer. Further track-bearing layers occur 170 cm and 10 cm above the main hominin track-bearing layer. These tracks are visible on the cave ceiling or in the surrounding cliffs, and on exposed surfaces east and west of the cave entrance. Multiple tracks are also evident on the layer that forms most of the cave floor, 50–90 cm below the main track-bearing layer. The layers represent dune surfaces in the stratigraphic record which may not be separated by significant gaps in time. A variety of non-hominin track types are represented, including tracks of elephant, various sizes of even-toed ungulate, large carnivore, giant Cape horse, probable juvenile ostrich, and small, unidentifiable tracks. Elsewhere along the beach and in the cliffs above there are further fossil vertebrate trackways, including a faint rhinoceros trackway, smaller equid tracks and buffalo trackways, as well as numerous invertebrate traces. Many of the tracks along the intertidal zone are periodically covered by sand, and are only exposed when the beach is relatively sediment-starved.

## Discussion

We suggest a likely age of this trackway to MIS 5c/~90 ka. We note that OSL dating is required to resolve the chronology of the site. The analysis of geological samples in thin section showed that the cement is blocky- to drusy calcite spar. This is interpreted to suggest diagenesis in the vadose zone^43,44^, through percolation of meteoric water which led to dissolution of calcium carbonate components of the sediment. The presence of one generation of calcium carbonate cement in these deposits (blocky calcite spar, Fig. 6b,f), which is indicative of cementation in the meteoric vadose zone, suggests that these deposits have not been re-submerged since deposition and lithification. There is no subsequent marine influence, and transmitted light petrography suggested that ~30% of the pores are unfilled. This is consistent with the overall situation recorded for the Cape south coast during the Pleistocene from existing literature^21,28,45^ and the modern coast-parallel wind regime^46^, with east to southeast winds dominant in the summer, and stronger winds from the westerly quadrant prevailing in the winter.

A further argument for the preferred younger age, is that at 90 ka the coastline was ~2 km distant^11,47–49^, compared to the MIS 5e shoreline which was at a point in excess of 6 m above present sea level^27,29,33^. At the time of MIS 5e, the level of the cliff containing the trackways (presently ~3 m above Mean Sea Level) would have been submerged by high sea levels.

Local^29,34,50^ and global^51^ studies have shown that during the Late Pleistocene, sea level did not reach the elevation of MIS 5e, nor did it near this elevation. The Holocene Highstand^52,53^ was likely associated with a 2–3 m higher-than-present sea level, but based on carbonate diagenesis its influence was not evident in the rock record at our study site. From MIS 5e, sea level retreated towards MIS 5c (which is the depositional age suggested here) with an associated shoreline ~ 2 km distant, when considering the local offshore bathymetry^48^. During MIS 5c, sea level was retreating and coastal dunes were likely to be migrating onto the now submerged coastal plain.

Tuttle’s criteria^17^ are met at the tracksite we describe, with the sole exception that the best preserved tracks exhibit three lateral digits, not four (e.g. Fig. 7a,b). This difference, we contend, is a function of preservation, not trackmaker anatomy.

The nine tracks on the northern surface that contain digital impressions were made by bipedal humans. The preservation of high definition features such as digital impressions suggests a relatively firm, possibly damp substrate, and rapid infilling of the track-bearing surface. The tracks on the southern surface do not unequivocally exhibit digit impressions. When viewed in isolation these approximate a hominin footprint morphology, but lack some of the more diagnostic hominin footprint characteristics to allow unequivocal identification. However, their occurrence in the same bedding plane, and with similar downslope bearing as the tracks on the northern surface (in some cases probably forming extensions of those trackways) strongly suggests a hominin origin, evidently from the same group of individuals or perhaps others in the group. This is likely an example of the influence of a change in the condition of the substrate from the northern surface to the southern surface on this bedding plane. Increased erosion of tracks nearer the cave mouth may also be a factor in downgrading track preservation.

The tracks seen in sagittal section in the east wall of the cave have a pace length and outline consistent with hominin tracks, and have a similar orientation as the other hominin tracks. Unequivocal confirmation of their hominin origin could be obtained by future excavation of the surrounding rock layers to reveal these natural casts *in situ*. The limited information that can be obtained for the tracks on the lower layer suggests a morphology intermediate between the tracks of the northern and southern surfaces of the main track-bearing layer.

The occurrence of tracks in both lateral walls of the cave, and at the junctions of the ceiling with both lateral walls and with the inner (north) wall, suggests that more tracks and trackways could easily be exposed. These tracks may be preserved with superior detail compared with those currently visible, many of which are partially eroded. Ideally the area between the northern and southern surfaces of the main track-bearing layer could be exposed, to yield one continuous track-bearing surface.

The most obvious trackway feature is the right-left sequence of large tracks. The pace length between tracks 4 and 8 is 85 cm. Track 11 is a poorly preserved right track. It probably represents a third track in this trackway, although it is not perfectly aligned with track 4 and track 8. The putative pace length between track 8 and track 11 is 66 cm. The distance between track 8 and the edge of the surface is 105 cm. Either way, a short-long (variable or alternating) gait pattern is evident. Such a gait may be employed when moving fast down a dune slope while heel planting, thereby aiding stability.

Another inferred hominin trackway is evident in the east wall of the cave (tracks 28 A, 28, 29, 30). The measured distances between these tracks, which appear in sagittal section, are 160 cm, 144 cm, 73 cm. The edge of a further track midway between tracks 28 and 29 is possibly present, yielding further pace lengths of 72 cm and 72 cm. If this is indeed a trackway, then these measurements indicate a different and more consistent gait pattern with a stride length of 144–160 cm and a pace length where measurable of 72–73 cm. These tracks also exhibit deep heel impressions, and are of similar length to many of the tracks seen on the northern surface. If an inferred pace length of ~73 cm is used, a number of plausible trackways become apparent on the northern surface, and some of these could extend into the tracks on the southern surface. However, this inference remains speculative, and until a larger track surface is exposed various interpretations are possible. Nonetheless, noting the strongly unimodal orientation, our interpretation is that there were probably multiple trackmakers. Other explanations for a unimodal orientation include repeat visits with similar bearing within a short time interval, and landscape constraints (although such constraints are improbable on dune surfaces).

Detailed analysis of human track morphology comes from Holocene tracks on level surfaces in the Namib Desert^54^ and from studies on habitually barefoot subjects on level surfaces in Kenya^55^. However, the applicability of these studies in analysing the gaits of individuals who made tracks on the downslope of a 20° dune surface is likely limited. Research on characteristic track and gait features of humans travelling down dune slopes would aid in the interpretation of the site.

Inferences have been made on human foot morphology from hominin tracksites^14,55^, and human behaviour, including that of groups^56^. Some of these inferences have been disputed^15^. A morpho-classificatory and morphometric approach, of the kind applied by Citton *et al*.^57^, in the Grotta della Basura tracksite in Italy, may be useful at the site we describe here, in order to estimate the number of trackmakers. Such studies would best be performed once further tracks have been exposed.

The limitations and complexities of estimating stature, velocity, mass and other measurements from track data have been described by Bennett and Morse^14^. Stature inferences from footprint dimensions were made by Roberts^19^ for the Nahoon and Langebaan tracks, using a formula (footprint length ×6.67) derived from global mean data of Mietto *et al*.^58^. Roberts thereby derived height estimates of 128.06 cm for the Nahoon trackmaker and 152.07 cm for the Langebaan trackmaker. Applying this formula to the tracks we describe here yields estimates of 153.4 cm for the largest tracks and ~116 cm for the smaller tracks.

Roberts^19^ noted a short pace length for the Nahoon and Langebaan trackways, and concluded that this probably was related to the difficulty of negotiating sloping and unstable surfaces. He noted a pace length of 33.04 cm for the Nahoon tracks, where the slope of the dune face was estimated at ~17°, and of 50 cm for the Langebaan tracks, where the slope of the dune face was estimated at ~15°. The pace lengths we describe of 75 cm, 85 cm, and possibly greater than 105 cm, on a dune slope of equivalent or slightly greater angle, imply a more rapid trackmaker velocity, and may be consistent with a running gait. In our view, sufficient information is not yet available to yield a velocity estimate.

Dating studies above and below the hominin track horizon would allow this site to be placed with greater certainty within the context of the hominin track record, and its temporal relationship with the other southern African Pleistocene sites to be determined. We used an approach to reach a ~90 ka age estimate for the hominin tracks which considered carbonate diagenesis and a careful comparison with known sites in the area.

Placed in a global context, unusual features of the tracksite we describe include:

- occurrence in aeolianites (shared by the Nahoon and Langebaan sites)
- deep heel impressions and downslope sediment rims
- tracks made on an angled slope (shared with, *inter alia*, the Roccamonfina Volcano, Nahoon and Langebaan sites)
- unimodal orientation
- natural cast tracks (shared with the Nahoon site)
- confined space in a small cave, with tracks on a ceiling creating documentation challenges.

The Nahoon and Langebaan Pleistocene hominin tracksites proved the potential for coastal aeolianites to preserve such features. However, when compared with other hominin tracksites, they have been described as being poorly preserved^13^. Bennett and Morse^14^ note that some authorities have questioned the human origin of the Langebaan tracks, while acknowledging that it remains the most likely interpretation. Nonetheless these sites have been regarded as important in the record of hominin ichnology, as they filled a substantial gap in the hominin track record.

Uncertainty about the age of the Engare Sero site in Tanzania appears to have been resolved, and a date of ~19 ka has been reported^59^. If the attribution to *Homo neanderthalensis* of the Vârtop Cave tracksite is accepted^60^, then without the South African sites there are no tracksites that can be attributed to *Homo sapiens* before 46 ka. However, the attribution of the Langebaan and Nahoon tracks to *Homo sapiens* predated the identification of *Homo naledi* as a Pleistocene inhabitant of southern Africa. Until reliable criteria are developed to distinguish *Homo naledi* tracks from *Homo sapiens* tracks, we contend that both should be considered as plausible or at least possible trackmakers at Late Pleistocene hominin tracksites in southern Africa. However, *Homo naledi* has a limited reported spatial distribution (more than 1000 km from the site we describe) and a limited temporal distribution, with an estimated minimum age of 236 ka^26^. It therefore seems reasonable to consider *Homo sapiens* as an increasingly more probable trackmaker with progressively younger tracksites such as the site we describe on the Cape south coast.

The only three known hominin tracksites from the age in which cognitively modern humans emerged are from southern Africa. When compared with the frequency of occurrence of other tetrapod track morphotypes, the frequency of hominin track occurrences may reflect population density, or at least frequency of activity in coastal dune settings.

Full documentation of aeolianite tracks would require sub-marine studies, as most of the suitable deposits are currently under water on the continental shelf. Globally, coastal aeolianites occur mostly between latitudes 20° and 40°, predominantly in the southern hemisphere along the coast of Australia and the southern and eastern coast of South Africa^40^.

## Conclusions

The Cape south coast hominin tracksite reported here represents the largest and best preserved known archive of southern African Late Pleistocene human tracks to date, and adds to the knowledge obtained from the Nahoon and Langebaan sites. This site is located on the Southern Coastal Plain within an area of profound significance for the emergence of modern humans. While *Homo naledi* cannot be excluded as a possible trackmaker, the current estimated minimum age for *Homo naledi* of 236 ka, along with the limited spatial distribution reported for this taxon, makes it more plausible that the tracks were made by *Homo sapiens*. The association of hominin tracks with those of other tetrapods helps us understand Pleistocene coastal dune palaeoecology.

Exposure of further tracks at this site is desirable, with the objective of interpreting behavioural evidence with greater confidence, and clarifying hominin group size and downslope locomotor behaviour. A gait and speed analysis of habitually barefoot humans moving down dune slopes would aid this understanding. To reach these goals, we need not only to expose further tracks at the site described, but to monitor the exposure of further tracksites and document them rapidly with high quality methods.

The current best age estimate of ~90 ka needs to be refined through dating studies from above and below the hominin track horizon. A comprehensive study of all African aeolianite exposures is needed to better determine the significance of the southern African hominin sites within the global hominin track record. Ideally such work would include comprehensive documentation of all associated tracks and their palaeobiological significance.

## Electronic supplementary material

Supplementary information
