# Supplementary material for: A New Pleistocene Hominin Tracksite from the Cape South Coast, South Africa
Source: Sci Rep. 2018 Feb 28;8:3772. doi: 10.1038/s41598-018-22059-5 (PMC5830700; doi:10.1038/s41598-018-22059-5)
Supplement: Supplementary file 1 — Supplementary information [file 41598_2018_22059_MOESM1_ESM.pdf]

A NEW PLEISTOCENE HOMININ TRACKSITE FROM THE CAPE SOUTH COAST, SOUTH AFRICA.

CHARLES W. HELM, RICHARD T. MCCREA, HAYLEY C. CAWTHRA, MARTIN G. LOCKLEY, RICHARD M. COWLING, CURTIS W. MAREAN, GUY H.H. THESEN, TAMMY S. PIGEON & SINÈAD HATTINGH.

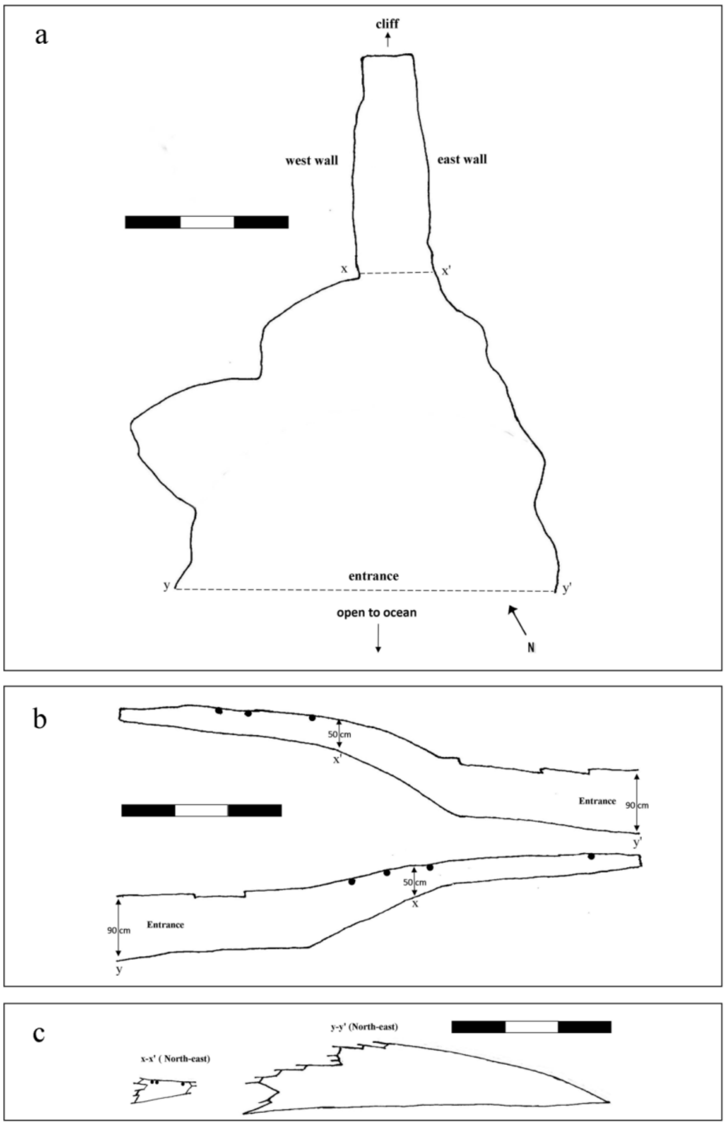

Supplementary Figure S1. Cave survey compiled by co-author SH. Scale bars are in metre intervals. Dots below the ceiling represent tracks. **(a)** floor plan. **(b)** sections of east wall (top) and west wall (bottom). **(c)** cross sections.

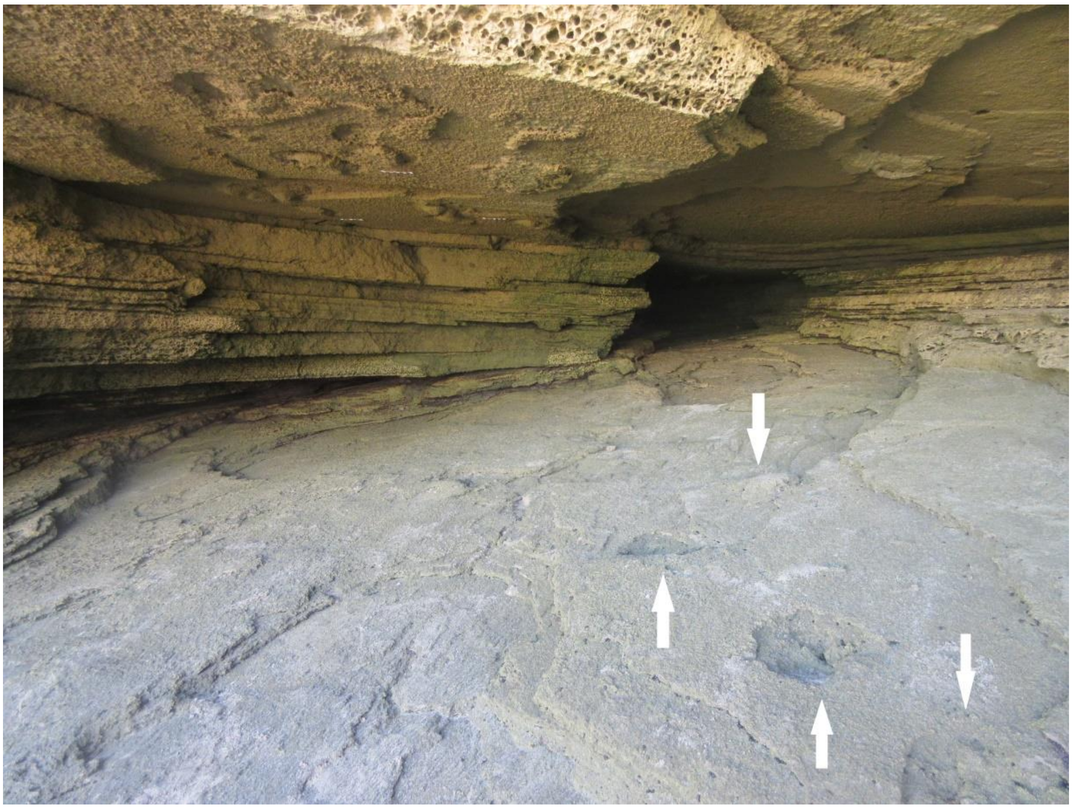

Supplementary Figure S2. On entering the cave, the southern hominin track-bearing surface can be seen on the ceiling (with 10 cm scale bars). The northern surface is within the dark section of the cave. Arrows indicate carnivore tracks on the cave floor (photograph by senior author CH).

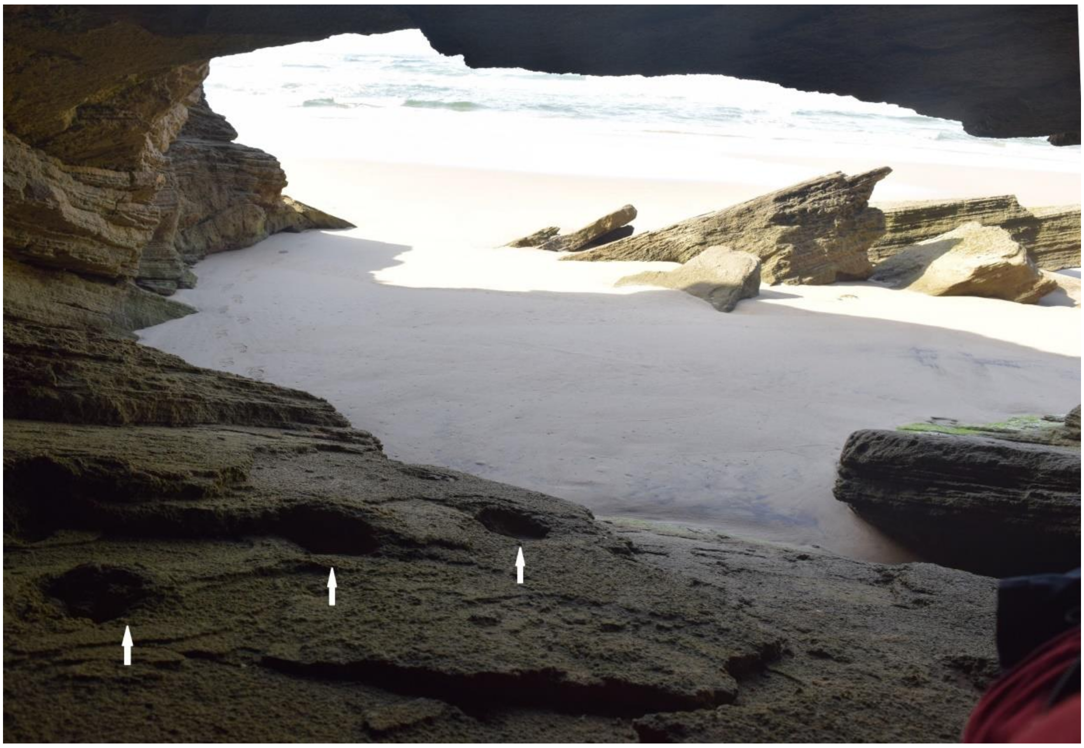

Supplementary Figure S3. View of intertidal zone and Indian Ocean from within the cave. Arrows indicate carnivore tracks on the cave floor (photograph by senior author CH).

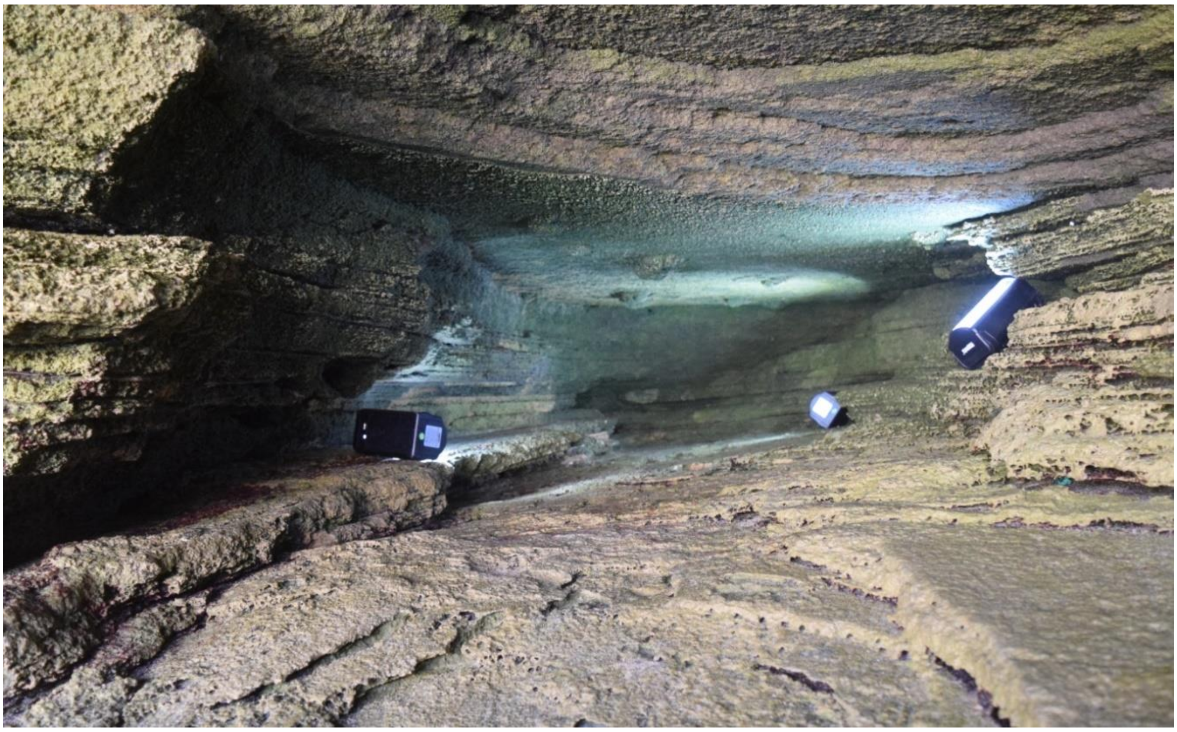

Supplementary Figure S4. The inner part of the cave **-** the northern hominin track-bearing surface, illuminated by three Magneto LED lanterns, forms the cave ceiling (photograph by senior author CH).

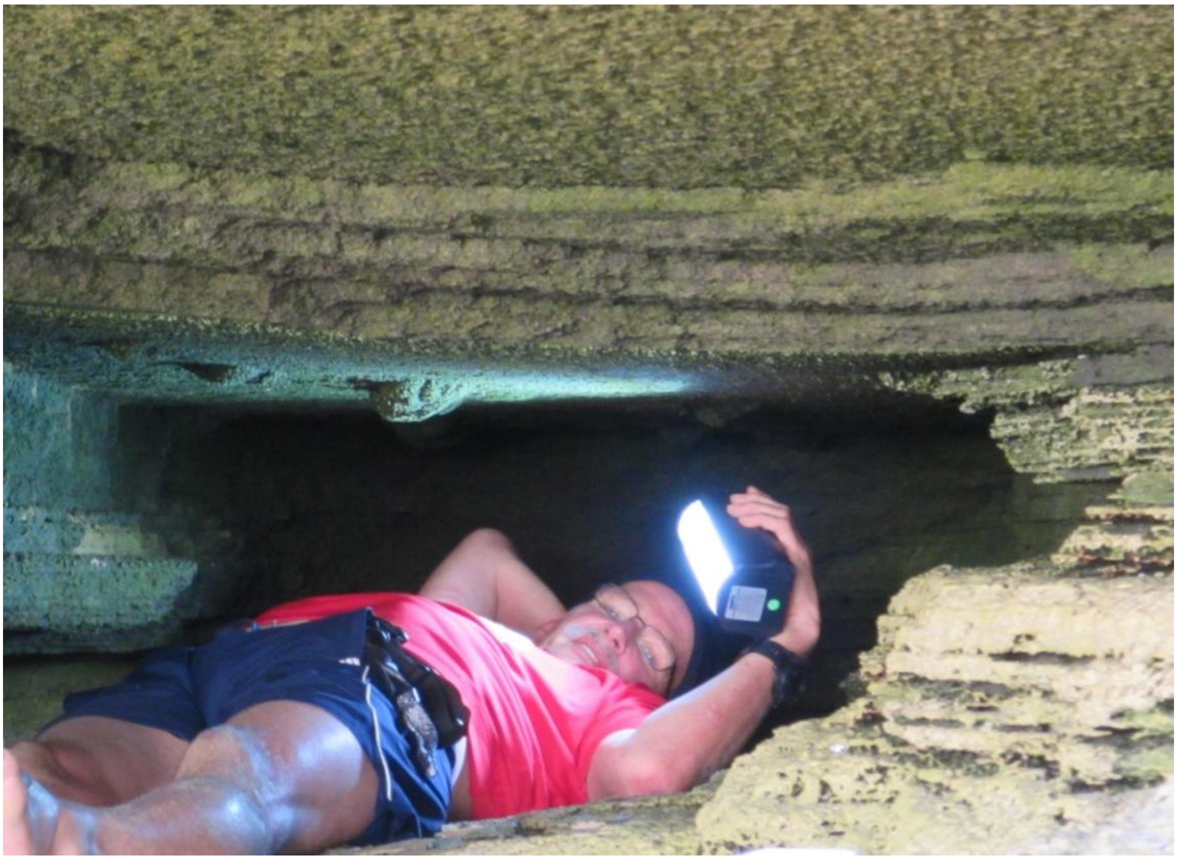

Supplementary Figure S5. The northern hominin track-bearing surface is on the ceiling of the confined inner part of the cave (photograph of senior author CH by co-author GT).
